# Supplementary material for: Genetic Diversity of Domestic Cat Hepadnavirus in Southern Taiwan
Source: Viruses. 2023 Oct 20;15(10):2128. doi: 10.3390/v15102128 (PMC10612041; doi:10.3390/v15102128)
Supplement: Supplementary file 1 [file viruses-15-02128-s001.zip › viruses-2664925-supplementary.pdf]

Supplemental Figure S1. Cluster heatmap showing the pairwise identity of complete genomes of DCH collected from Taiwan and available sequences from GenBank. Sequence alignment (ClustalW), clustering (neighbor joining tree), and pairwise sequence identity calculations were performed in SDTv1.2.

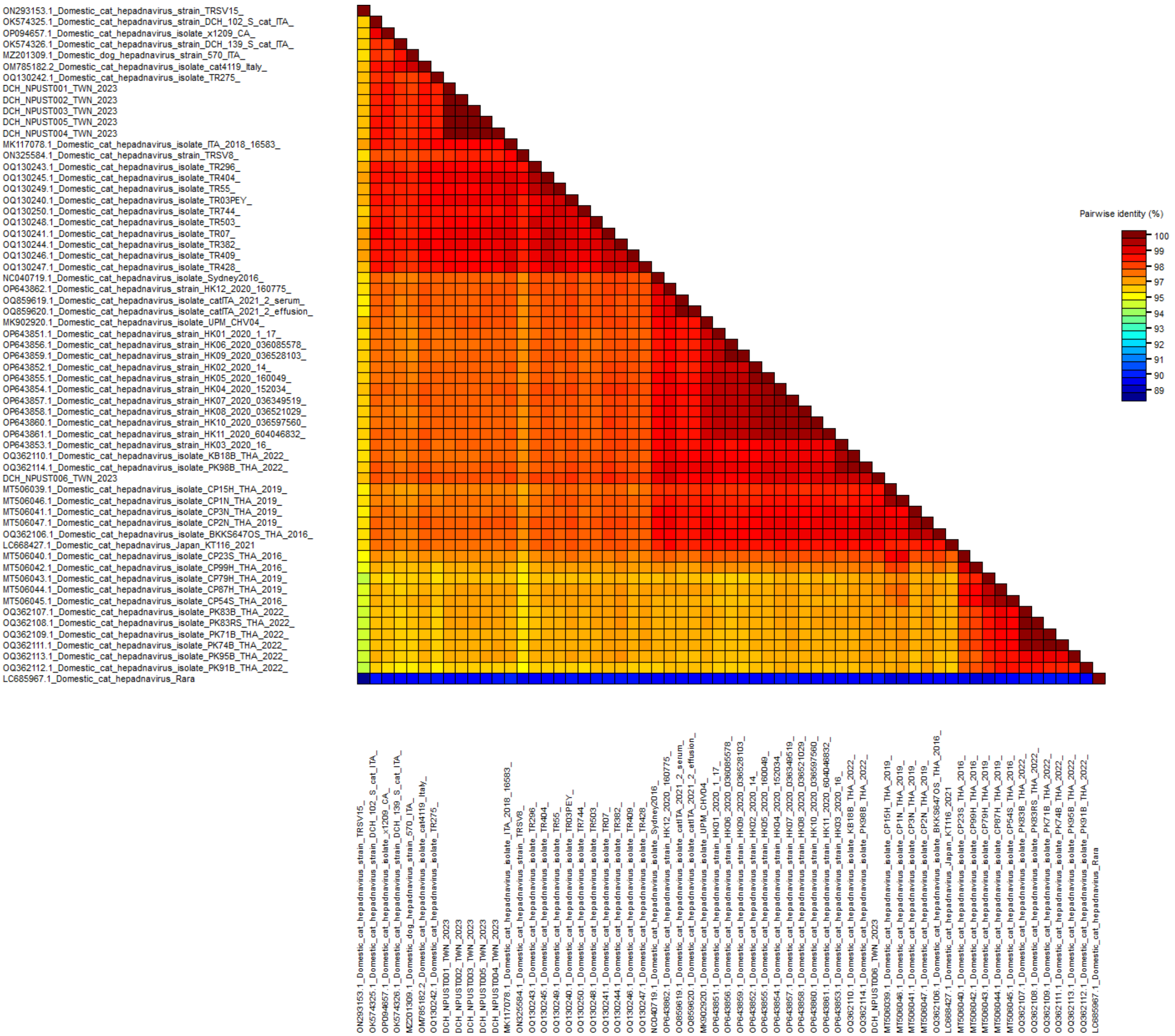

Supplemental Figure S2. Alignment the preS1 amino acid sequences showing high conservation among DCH strains.

A. preS1

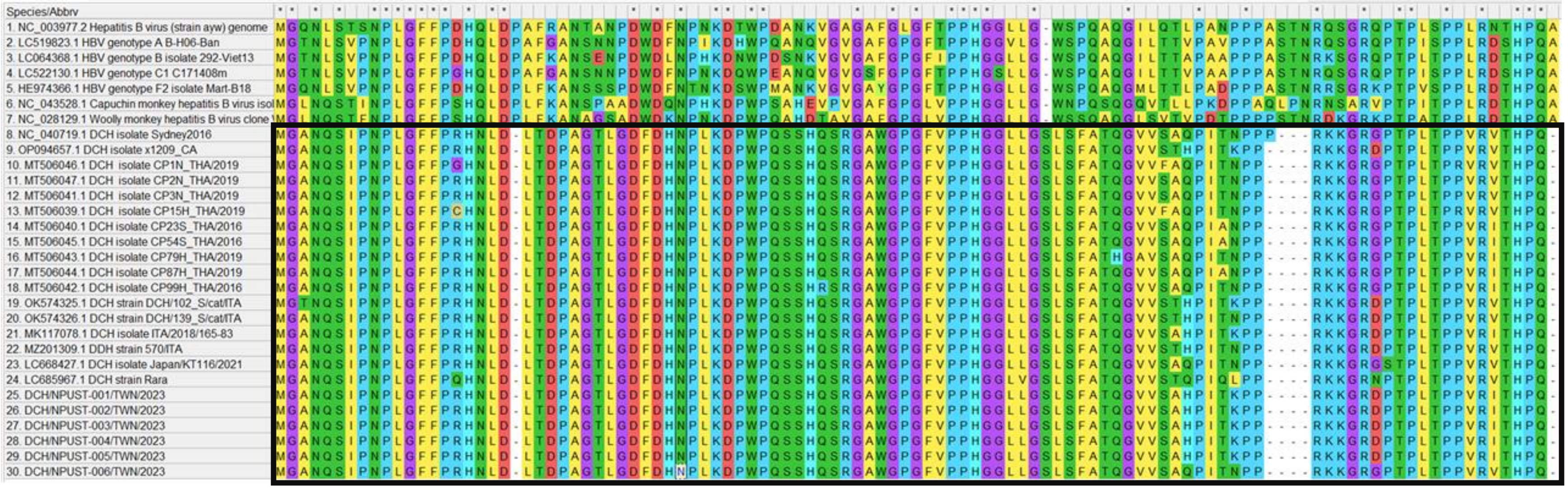

Supplemental Figure S3. Comparison of the phylogenetic trees generated from a previous report identifying Clades A1 and A2 under Genotype A and the updated tree that included more recently reported strains, particularly from Türkiye and Taiwan. Previously identified members of Clade A2 were distributed into separate branches instead of one robustly supported monophyletic clade.

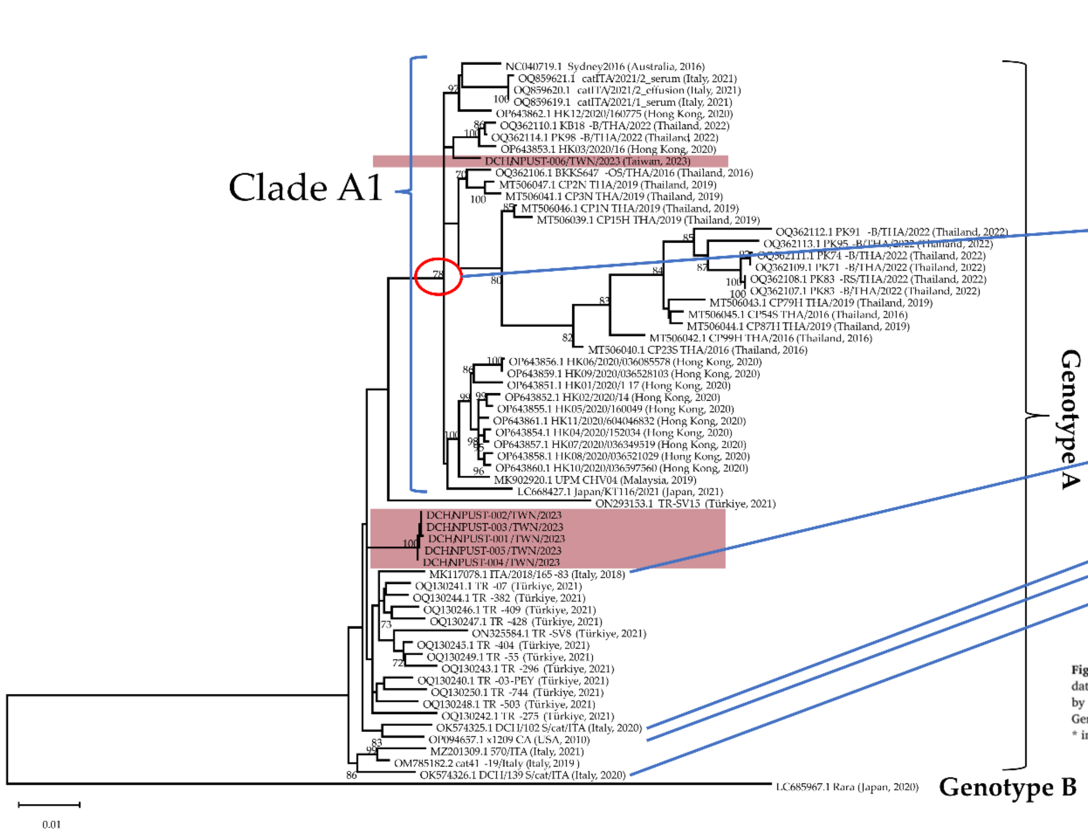

Figure 1 (from the main text)

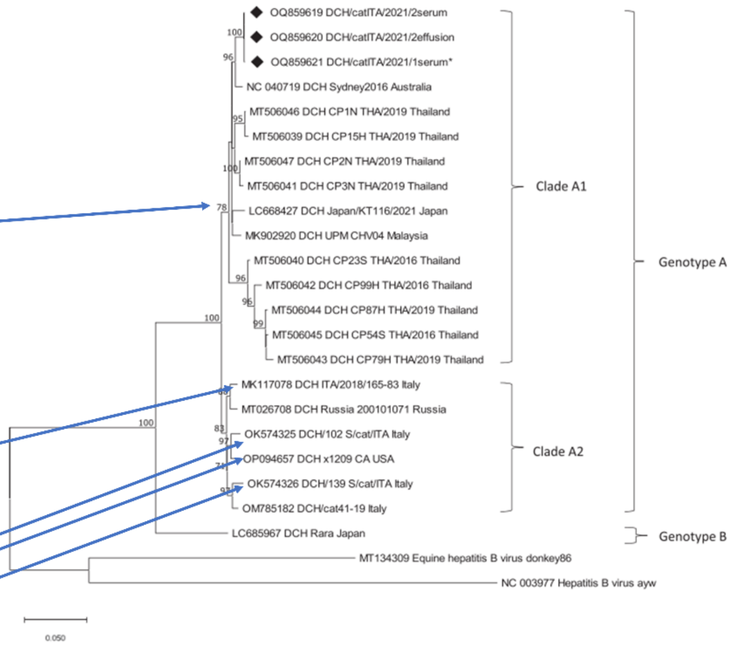

Fig. 1. Phylogenetic tree generated with maximum likelihood analysis. Whole genome sequences from domestic cat hepadnavirus (DCH) retrieved from GenBank databases and the DCH obtained in this study were used. Mammalian orthohepadnaviruses whole genome sequences were used as outgroup. Sequences are indicated by GenBank accession number (available at www.ncbi.nlm.nih.gov/pubmed/) name of the strain and geographical origin. Distances were computed using the General Time Reversible model using MEGA v.11. Bootstrap values above 70% are shown. The black diamonds indicate the sequences obtained in this study; * indicates partial whole genome sequence.

From Ratti, et al. 2023
(https://doi.org/10.1016/j.vetmic.2023.109828)

Supplemental Figure S4. Phylogenetic trees were constructed using the protein sequences for (**a**) polymerase, (**b**) surface, (**c**) core, and (**d**) X proteins of DCH strains from Taiwan and available sequences from GenBank. The tree was created by constructing a maximum likelihood tree with the best fit and chosen model according to the Bayesian information criterion using IQTREE v.2.2.2.3. Bootstrap values above 70% are shown. The scale bar indicates the number of substitutions per site.

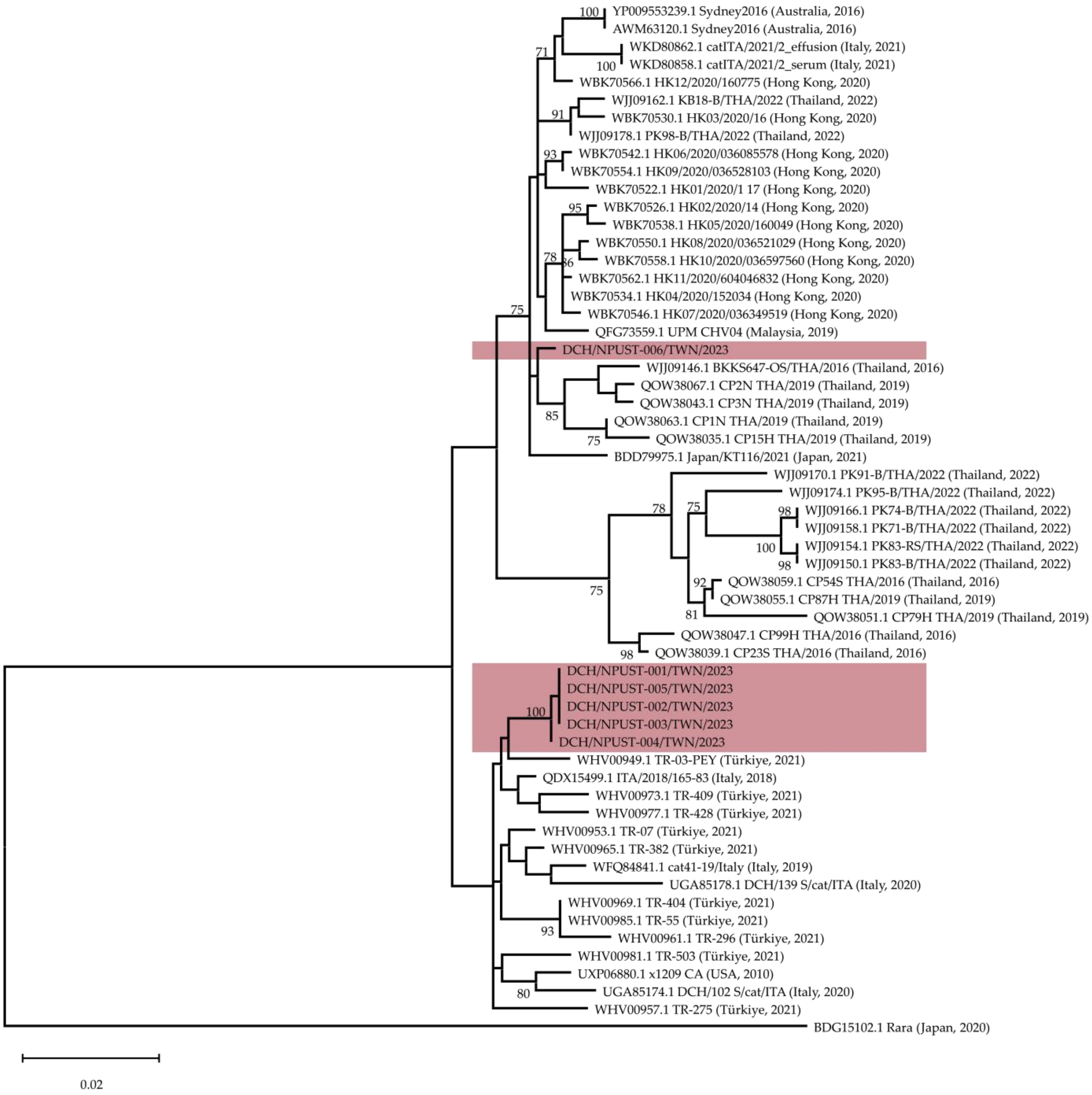

(**a**) Polymerase protein

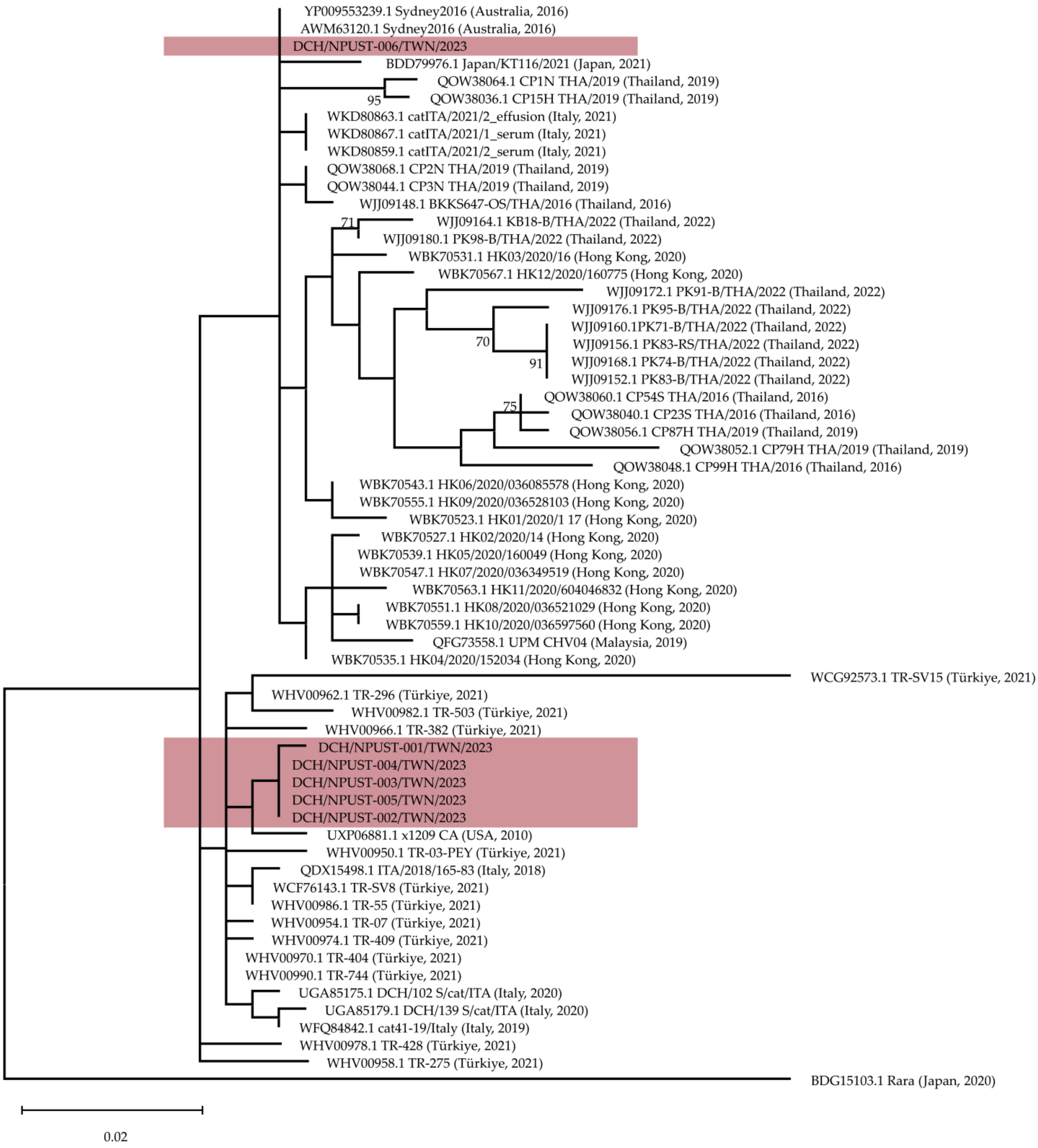

(**b**) Surface protein

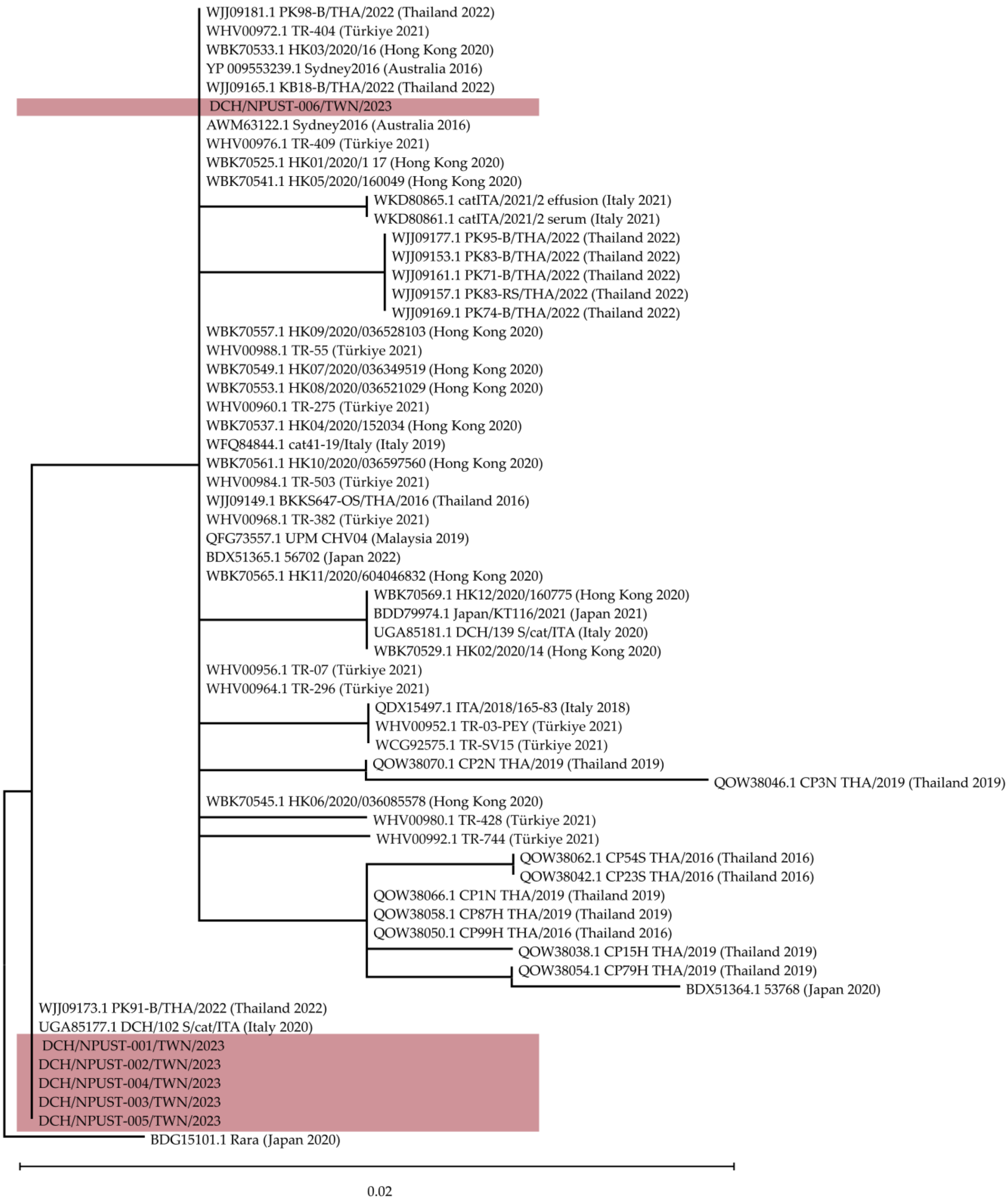

(**c**) Core protein

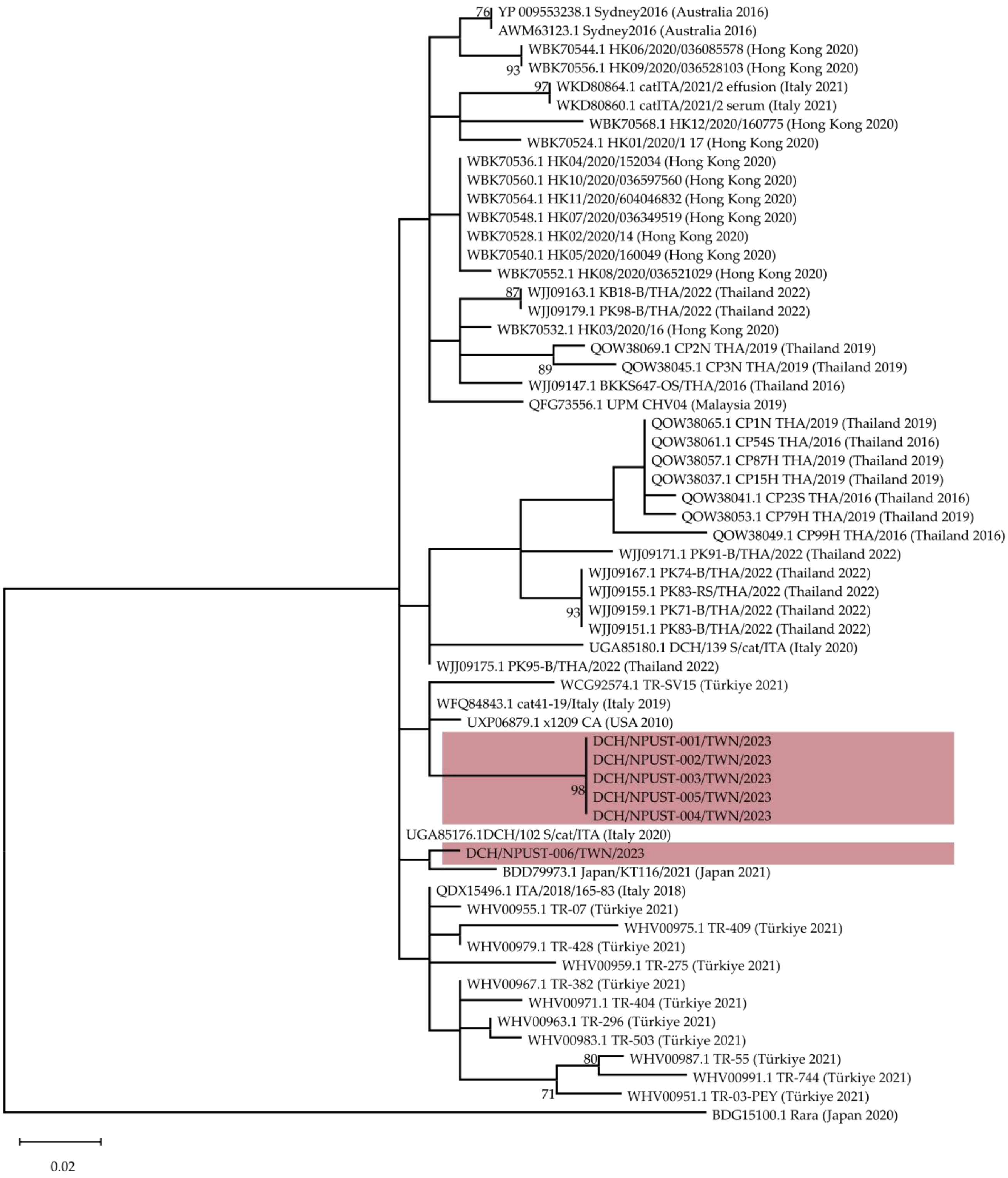

76
YP 009553238.1 Sydney2016 (Australia 2016)
AWM63123.1 Sydney2016 (Australia 2016)
WBK70544.1 HK06/2020/036085578 (Hong Kong 2020)
93
WBK70556.1 HK09/2020/036528103 (Hong Kong 2020)
97
WKD80864.1 catITA/2021/2 effusion (Italy 2021)
WKD80860.1 catITA/2021/2 serum (Italy 2021)
WBK70568.1 HK12/2020/160775 (Hong Kong 2020)
WBK70524.1 HK01/2020/1 17 (Hong Kong 2020)
WBK70536.1 HK04/2020/152034 (Hong Kong 2020)
WBK70560.1 HK10/2020/036597560 (Hong Kong 2020)
WBK70564.1 HK11/2020/604046832 (Hong Kong 2020)
WBK70548.1 HK07/2020/036349519 (Hong Kong 2020)
WBK70528.1 HK02/2020/14 (Hong Kong 2020)
WBK70540.1 HK05/2020/160049 (Hong Kong 2020)
WBK70552.1 HK08/2020/036521029 (Hong Kong 2020)
87
WJJ09163.1 KB18-B/THA/2022 (Thailand 2022)
WJJ09179.1 PK98-B/THA/2022 (Thailand 2022)
WBK70532.1 HK03/2020/16 (Hong Kong 2020)
QOW38069.1 CP2N THA/2019 (Thailand 2019)
89
QOW38045.1 CP3N THA/2019 (Thailand 2019)
WJJ09147.1 BKKS647-OS/THA/2016 (Thailand 2016)
QFG73556.1 UPM CHV04 (Malaysia 2019)
QOW38065.1 CP1N THA/2019 (Thailand 2019)
QOW38061.1 CP54S THA/2016 (Thailand 2016)
QOW38057.1 CP87H THA/2019 (Thailand 2019)
QOW38037.1 CP15H THA/2019 (Thailand 2019)
QOW38041.1 CP23S THA/2016 (Thailand 2016)
QOW38053.1 CP79H THA/2019 (Thailand 2019)
QOW38049.1 CP99H THA/2016 (Thailand 2016)
WJJ09171.1 PK91-B/THA/2022 (Thailand 2022)
WJJ09167.1 PK74-B/THA/2022 (Thailand 2022)
WJJ09155.1 PK83-RS/THA/2022 (Thailand 2022)
93
WJJ09159.1 PK71-B/THA/2022 (Thailand 2022)
WJJ09151.1 PK83-B/THA/2022 (Thailand 2022)
UGA85180.1 DCH/139 S/cat/ITA (Italy 2020)
WJJ09175.1 PK95-B/THA/2022 (Thailand 2022)
WCG92574.1 TR-SV15 (Türkiye 2021)
WFQ84843.1 cat41-19/Italy (Italy 2019)
UXP06879.1 x1209 CA (USA 2010)
DCH/NPUST-001/TWN/2023
DCH/NPUST-002/TWN/2023
DCH/NPUST-003/TWN/2023
98
DCH/NPUST-005/TWN/2023
DCH/NPUST-004/TWN/2023
UGA85176.1DCH/102 S/cat/ITA (Italy 2020)
DCH/NPUST-006/TWN/2023
BDD79973.1 Japan/KT116/2021 (Japan 2021)
QDX15496.1 ITA/2018/165-83 (Italy 2018)
WHV00955.1 TR-07 (Türkiye 2021)
WHV00975.1 TR-409 (Türkiye 2021)
WHV00979.1 TR-428 (Türkiye 2021)
WHV00959.1 TR-275 (Türkiye 2021)
WHV00967.1 TR-382 (Türkiye 2021)
WHV00971.1 TR-404 (Türkiye 2021)
WHV00963.1 TR-296 (Türkiye 2021)
WHV00983.1 TR-503 (Türkiye 2021)
80
WHV00987.1 TR-55 (Türkiye 2021)
WHV00991.1 TR-744 (Türkiye 2021)
71
WHV00951.1 TR-03-PEY (Türkiye 2021)
BDG15100.1 Rara (Japan 2020)
0.02

(**d**) X protein
